# Real‐world insights on switching aflibercept dosage for enhanced outcomes in age‐related macular degeneration (RISE‐AMD): Safety data

**DOI:** 10.1111/aos.70160

**Published:** 2026-05-27

**Authors:** Mary Graciya Karunainathan, Celine Lee, Kaweh Daniel Zohalinejad, Mads Odgaard Mæng, Nanna Würtz Fabrin, Sigurrós Jónsdóttir, Anne Kjærsgaard Hassing, Søren Kveiborg Yde, Charles C. Wykoff, Henrik Vorum, Yousif Subhi, Marie Ørskov, Lasse Jørgensen Cehofski

**Affiliations:** ^1^ Department of Clinical Medicine Aalborg University Aalborg Denmark; ^2^ Department of Ophthalmology Aalborg University Hospital Aalborg Denmark; ^3^ Retina Consultants of Texas Retina Consultants of America Houston Texas USA; ^4^ Department of Ophthalmology Rigshospitalet Copenhagen Denmark; ^5^ Department of Clinical Research University of Southern Denmark Odense Denmark

**Keywords:** adverse events, aflibercept 8 mg, age‐related macular degeneration, Eylea 8 mg, intraocular inflammation, intraocular pressure

## Abstract

**Purpose:**

To evaluate real‐world safety experience with aflibercept 8 mg in a large case series of patients with neovascular age‐related macular degeneration (nAMD).

**Methods:**

Due to a high proportion of patients on short treatment intervals, patients with nAMD at a Danish tertiary retina clinic were systematically switched to aflibercept 8 mg. Safety data from May 2024 to October 2025 were obtained through a retrospective review of medical records by an experienced retina specialist and an additional reviewer. Symptomatic intraocular pressure (IOP) increase was defined as any symptom (e.g. pain) in conjunction with IOP > 21 mmHg.

**Results:**

In total, 8675 intravitreal injections of aflibercept 8 mg were administered in 1429 eyes with nAMD, including 139 glaucomatous eyes. A total of 25 AEs were reported (2.88 per 1000 injections). Intraocular inflammation (IOI) was reported in 11 eyes (1.27 per 1000 injections), with 10 eyes being managed with topical dexamethasone, while one case required vitrectomy. Symptomatic IOP increase was observed in 10 cases (1.15 per 1000 injections). The occurrence of symptomatic IOP increase did not differ between glaucomatous and non‐glaucomatous eyes (*p* = 0.24). Two cases of bacterial endophthalmitis were registered. No case of retinal vasculitis or retinal vascular occlusion was observed.

**Conclusion:**

Incidence rates of symptomatic IOP increase and IOI were generally low. Despite a large glaucoma cohort, the higher incidence rate of symptomatic IOP increase in glaucomatous eyes did not reach statistical significance.

## 1 INTRODUCTION

Age‐related macular degeneration (AMD) is a major cause of severe vision loss globally and it is estimated to affect 288 million individuals in 2040 (Flaxel et al., 2020; Wong et al., 2008, 2014). The anti‐VEGF agent, aflibercept 8 mg, was introduced following the PULSAR (Lanzetta et al., 2024) phase 3 randomized, double‐masked trial that demonstrated robust durability with 83% of the patients maintaining intervals ≥12 weeks at week 48. Aflibercept 8 mg is a novel formulation that is administered at a higher concentration and volume than aflibercept 2 mg (70 μL of 114.3 mg/mL vs. 50 μL of 40 mg/mL). Despite favourable durability outcomes in clinical trials and some early real‐world datasets, adverse events (AEs) related to the higher concentration and volume remain clinical concerns. Although the PULSAR trial (Lanzetta et al., 2024) reported similar safety profiles for aflibercept 8 mg and 2 mg, limited comprehensive data from real‐world clinical practice regarding the AE profile may delay clinician choice to switch to the higher dose. The existing real‐world data on aflibercept 8 mg predominantly comprise studies of small sample sizes (Rebsdorf et al., 2025). High incidence rates of intraocular inflammation (IOI) of 36.7–85.7 cases per 1000 injections have previously been reported in case series of 35–41 eyes (Binder et al., 2025; Matsumoto et al., 2024). Although case series have provided insights into IOI associated with aflibercept 8 mg, there is a need for systematic, large‐scale, high‐quality data generated from consecutive patients in routine clinical practice.

The Real‐world Insights on Switching Aflibercept Dosage for Enhanced Outcomes in Age‐related Macular Degeneration (RISE‐AMD) initiative was launched to reduce the short‐interval treatment burden. The project was a single centre initiative where the entire population of patients with neovascular age‐related macular degeneration (nAMD) being cared for at a tertiary Danish ophthalmology clinic was systematically switched to aflibercept 8 mg regardless of disease control. Herein, we report on large‐scale safety data following 8675 consecutive intravitreal injections of aflibercept 8 mg.

## 2 METHODS

### 2.1 Study population and study design

Data were collected retrospectively beginning from the treatment initiation of aflibercept 8 mg. The retrospective evaluation of the RISE‐AMD safety data was approved by the North Denmark Region (Project ID: K2024‐213) and adhered to the tenets of the Helsinki Declaration. All data management was performed in accordance with the General Data Protection Regulation (GDPR). As part of the RISE‐AMD initiative, all patients with nAMD at the Department of Ophthalmology, Aalborg University Hospital, Denmark, were systematically switched to aflibercept 8 mg beginning in May 2024, except for a few eyes that were kept on faricimab or ranibizumab at clinicians' discretion. For the retrospective evaluation, patients with nAMD were identified through the Danish health registers by the ICD‐10 codes for AMD (DH353, DH353K, DH353J, DH353L) and intravitreal injection procedure codes (KCKD05 and KCKD05B). The personal identification number (CPR) of the identified patients was retrieved to access the respective medical records and optical coherence tomography (OCT) scans. All eyes with a correct nAMD diagnosis receiving aflibercept 8 mg were included. Eyes that were switched to aflibercept 8 mg, as well as treatment naïve eyes receiving aflibercept 8 mg as first‐line therapy were eligible for inclusion. Eyes that did not receive aflibercept 8 mg or had an incorrect diagnosis of nAMD were excluded.

All injections were administered through vial splitting, with each vial yielding two injections, as the centre was authorized to perform this procedure. Injections were performed in clinical suites. It was mandatory for the staff to wear a face mask and a surgical cap. Pre‐injection assessment for blepharitis was performed. Topical anaesthesia was induced with repeated instillation of tetracaine 1% eye drops followed by the instalment of topical povidone‐iodine 5%. Afterwards, povidone‐iodine 5% was applied to the eyelid margins and excess povidone‐iodine solution was used to cleanse the eyelid margins. It was mandatory for the operator to wear sterile gloves. An eyelid speculum was inserted and intravitreal injection was administered under sterile conditions in the inferotemporal quadrant 3.5 mm posterior to the limbus, unless any other site was specified. Bilateral injections were performed on the same day unless the patient specifically asked for injections on two separate days. Patients were instructed to monitor for symptoms suggestive of retinal detachment, bacterial endophthalmitis, IOI, or symptomatic intraocular pressure (IOP) increase. Comprehensive hygiene instructions were provided, including avoidance of eye rubbing, exposure to dusty environments, swimming, and any other potential sources of contamination.

Medical records for each patient were initially reviewed by one of the authors and subsequently reassessed by an experienced retina specialist. All data were entered into a secure platform (15.5.15, REDCap; Vanderbilt University, Nashville, USA). Baseline data were obtained, including sex, age, number of previous injections, anti‐VEGF agent prior to switch, pre‐existing glaucoma or ocular hypertension (OHT), and IOP‐lowering medications prescribed prior to the initiation of aflibercept 8 mg. All patients were managed under an observe‐and‐plan regimen (Subhi et al., 2024). AEs were registered at each follow‐up visit, which were categorized into the nearest pre‐defined time‐point (3‐, 6‐, or 12‐month follow‐up) from the initiation of aflibercept 8 mg. Any AEs were registered. Presence or absence of symptomatic IOP increase, IOI, endophthalmitis, retinal vasculitis, and retinal vascular occlusion was noted at every follow‐up visit. Additional information concerning aqueous or vitreous taps were registered in the event of any AE. IOP was routinely accessed with an iCare rebound tonometer (iCare Finland Oy) at each 6‐month follow‐up. Additional IOP measurements were obtained if the patient complained about symptoms. Symptomatic IOP increases observed at outpatient clinics or reported by ophthalmologists outside the referral centre were included. In cases where iCare tonometry measured an IOP > 21 mmHg, Goldmann applanation tonometry was performed. Symptomatic IOP increase was defined as any symptom (e.g. pain, blurred vision, or vision loss) occurring in conjunction with IOP > 21 mmHg.

### 2.2 Statistical analyses

Statistical analyses were performed in stata 19.5 [StataCorp, College Station, Texas, USA]. Descriptive statistics were used to summarize baseline characteristics. Numerical variables were presented as mean ± standard deviation. Categorical variables were presented as percentages. All AEs were calculated as incidence rate per 1000 injections. A chi‐square test was conducted to compare the risk of AEs between glaucomatous versus non‐glaucomatous eyes, as well as AEs in naïve versus switched eyes. Differences in the occurrence of AEs between the 3‐, 6‐, or 12‐month follow‐up visits were evaluated by a chi‐square test. Fisher's exct test was applied when the expected observation in one or more cells was lower than five. Normality was assessed by Shapiro–Wilk test. Paired *T*‐test was performed to evaluate changes in best corrected visual acuity (BCVA) before and after an event of IOI. A significance level of *p* < 0.05 was considered statistically significant.

## 3 RESULTS

Among the 2452 patients with a nAMD diagnosis, 1254 patients were excluded for not receiving aflibercept 8 mg, having an incorrect diagnosis of nAMD, or missing data (Figure 1). We identified 1429 eyes that received aflibercept 8 mg (Table 1) in a population of 1198 patients with nAMD (62.60% female and 37.40% male, average age 82.15 ± 7.30 years). In the study period between May 2024 and October 2025, a total of 8675 injections were administered in the population. A mean of 6.07 ± 2.80 aflibercept 8 mg injections were administered per eye. Among the 1429 eyes that received aflibercept 8 mg, 5.32% (*n* = 76) received 1–2 injections, 58.85% (*n* = 841) received 3–6 injections and 35.83% (*n* = 512) received > 6 injections of aflibercept 8 mg. A total of 1101 eyes were switched from other anti‐VEGF agents to aflibercept 8 mg, while 328 eyes were treatment naïve. The majority of the previously treated eyes were switched from aflibercept 2 mg (*n* = 963, 87.46%), while a smaller proportion were switched from faricimab (*n* = 81, 7.36%) or ranibizumab (*n* = 57, 5.18%). The cohort included 139 eyes with pre‐existing glaucoma (9.73%) and 8 eyes with pre‐existing OHT (0.56%) (Table 1).

**FIGURE 1:**
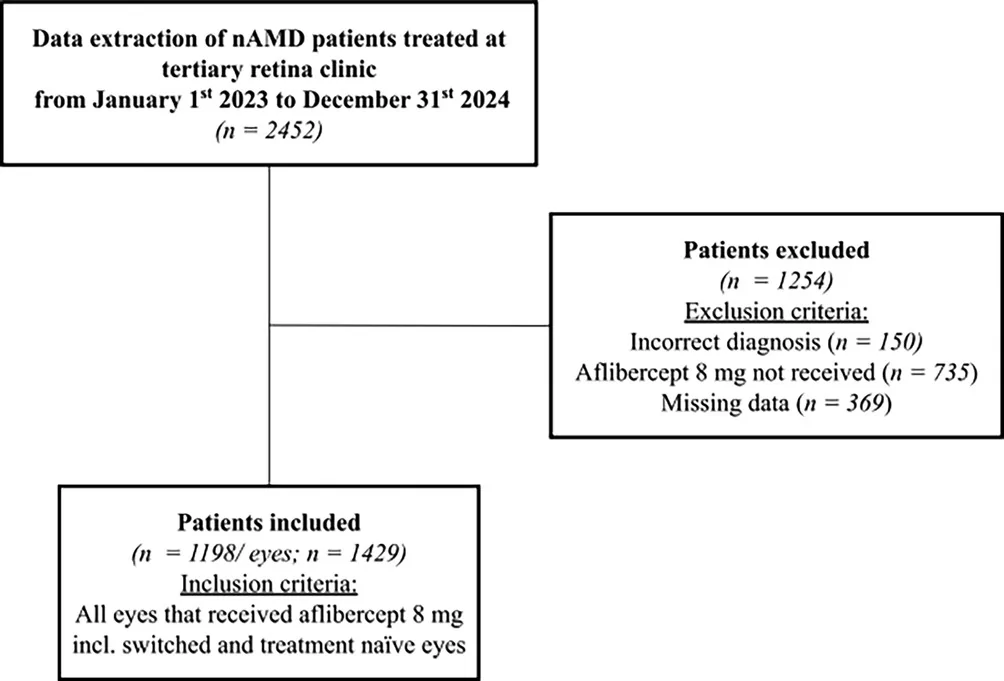
Inclusion flow‐chart.

**TABLE 1:** Study population.

Cohort overview	
Patients, *n*	1198
Age (mean)	82.15 ± 7.30
Sex	
Male, *n* (%)	448 (37.40)
Female, *n* (%)	750 (62.60)
Eyes in total, *n*	1429
Pre‐existing glaucoma, *n*	139
Pre‐existing OHT, *n*	8
Treatment naïve, *n*	328
Previously treated, *n*	1101
BCVA, ETDRS letters	65.59 ± 14.90
Average number of injections per eye before switch	22.49 ± 22.99
Most recent anti‐VEGF agent before switch	
Aflibercept 2 mg, *n* (%)	963 (87.46)
Faricimab, *n* (%)	81 (7.36)
Ranibizumab, *n* (%)	57 (5.18)
Total number of injections with aflibercept 8 mg	8675
Mean number of injections received, *n*	6.07 ± 2.80
1–2 injections received, *n* (%)	76 (5.32)
3–6 injections received, *n* (%)	841 (58.85)
> 6 injections received, *n* (%)	512 (35.83)

Out of the 8675 administered injections, 25 AEs were observed, corresponding to 2.88 AEs per 1000 injections (Table 2). IOI was reported in 11 eyes, corresponding to an incidence rate of 1.27 per 1000 injections. Symptomatic IOP increase was observed in 10 cases, corresponding to an incidence rate of 1.15 events per 1000 injections (Table 2). Two cases of bacterial endophthalmitis were reported, corresponding to 0.23 events per 1000 injections, with both cases having a positive culture of *Staphylococcus warneri*. These cases of endophthalmitis were reported 5.5 (±0.70) days after injection with aflibercept 8 mg. One case of retinal detachment was observed, corresponding to an incidence rate of 0.12 per 1000 injections. This event occurred 12 days after injection with aflibercept 8 mg and was managed with vitrectomy, with BCVA declining from 62 to 43 Early Treatment Diabetic Retinopathy Study (ETDRS) letters. Additionally, a single case of herpetic keratitis was observed but considered unrelated to aflibercept administration. In eyes with a symptomatic IOP increase (*n* = 10), the most reported symptom was blurred vision (*n* = 5), followed by pain (*n* = 4), complete visual loss (*n* = 3), and floaters (*n* = 1), with three eyes having more than one symptom.

**TABLE 2:** Reported AEs and their incidence rates.

	Eyes, *n*	Incidence rate per 1000 injections
Total number of adverse events	25	2.88
IOI	11	1.27
Symptomatic IOP increase	10	1.15
Endophthalmitis	2	0.23
Retinal detachment	1	0.12
Herpetic keratitis	1	0.12
Retinal vascular occlusion	0	0
Retinal vasculitis	0	0

The most reported AE was IOI (*n* = 11). On average, IOI occurred after 4.64 (±2.50) injections, with 18.18% of events occurring after the first injection and 50% within four injections of aflibercept 8 mg. IOI was on average reported 19 days after injection with aflibercept 8 mg. Ten out of the eleven IOI cases were managed with topical dexamethasone, while one case required vitrectomy due to dense vitritis that did not resolve on topical dexamethasone eye drops (Table 3). No statistically significant difference in BCVA before (62.45 ± 19.47 ETDRS letters) and after (64.64 ± 17.62 ETDRS letters) IOI was observed (difference − 2.18 ETDRS letters [95% CI; −10.00 to 5.64]; *p = 0.55*). After IOI, seven eyes were switched to ranibizumab and one eye was discharged due to disease inactivity. Three eyes remained on aflibercept 8 mg at the clinicians' discretion (Table 3). One eye had a history of recurrent uveitis with an IOI considered unrelated to aflibercept 8 mg, while the other two eyes were kept on aflibercept 8 mg due to significantly better durability than on previous agents, with one of the eyes being treated routinely with topical dexamethasone eye drops pre‐ and post‐injection. Recurrence of inflammation was not reported in any of the IOI cases.

**TABLE 3:** Post‐injection IOI.

Patient	Type of inflammation	Severity – anterior chamber cells	IOI management*	Post‐IOI treatment
1	Anterior uveitis	N/A	Topical	Ranibizumab
2	Anterior uveitis	0.5+	Topical	Ranibizumab
3	Anterior uveitis	3+	Topical	Aflibercept 8 mg
4	Anterior uveitis	1+	Topical	Ranibizumab
5	Anterior uveitis	N/A	Topical	Discharged due to inactivity in nAMD.
6	Anterior uveitis	0.5+	Topical	Aflibercept 8 mg
7	Combined anterior uveitis and vitritis	1+	Topical	Ranibizumab
8	Vitritis	0	Topical	Aflibercept 8 mg
9	Vitritis	0	Topical	Ranibizumab
10	Vitritis	0	Topical	Ranibizumab
11	Panuveitis	1+	Topical and vitrectomy	Ranibizumab

* Topical refers to topical dexamethasone 1 mg/1 mL eye drops.

There was no statistically significant difference in incidence rates of symptomatic IOP increase between glaucomatous eyes and non‐glaucomatous eyes (*p* = 0.24) (Figure 2). No AE was reported in eyes with OHT. Among the 15 eyes that were initiated on post‐injection IOP‐lowering medication, 12 eyes had a history of glaucoma or OHT (*p* < 0.001). Six eyes needed a volume reduction from 0.07 to 0.05 mL. Two of these eyes had pre‐existing glaucoma, and four required volume reduction due to symptomatic IOP increase. After volume reduction, there were no further events of symptomatic IOP increase in these eyes. In all cases, the volume reduction was initiated at the clinicians' discretion. One glaucomatous eye received selective laser trabeculoplasty after the treatment initiation with aflibercept 8 mg. This eye did not experience any AE and was therefore considered unrelated to injections with aflibercept 8 mg. One of the two glaucomatous eyes with symptomatic IOP increase had a history of filtration surgery and was treated with three IOP‐lowering medications before initiation of aflibercept 8 mg. The other glaucomatous eye with symptomatic IOP increase was a hyperopic eye, with a refraction of +5.75 dioptres, that had been managed with iridotomy before initiation of aflibercept 8 mg. This eye was not treated with IOP‐lowering medication at the time of switch. Both glaucomatous eyes were treated with an alpha‐adrenergic agonist before injection with aflibercept 8 mg as a precaution against IOP increase. The eye managed with iridotomy before initiation of aflibercept 8 mg additionally needed a volume reduction to 0.05 mL. There was no further symptomatic IOP increase noted in this eye.

**FIGURE 2:**
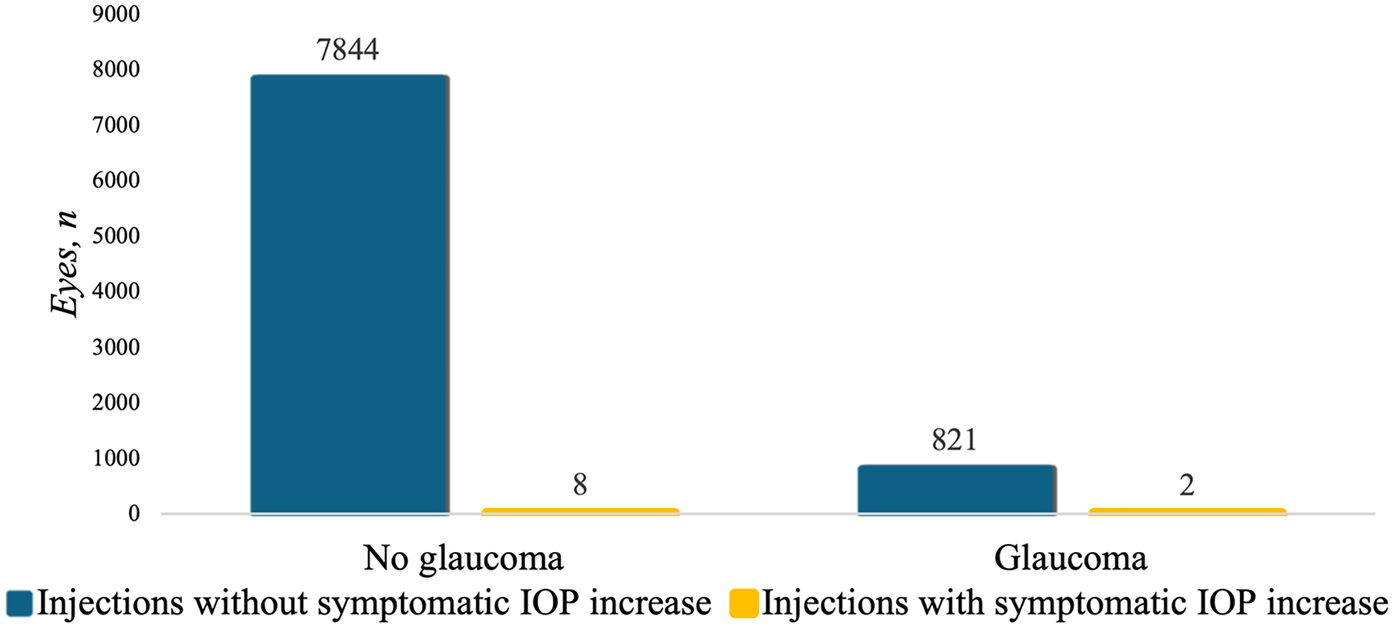
A total of 10 cases of symptomatic IOP increase were reported. The proportion of eyes with symptomatic IOP‐related AEs was not significantly higher for glaucomatous eyes (*p* = 0.24). In total, 139 glaucomatous eyes received 823 injections with two eyes developing symptomatic IOP increase following injection with aflibercept 8 mg.

Bacterial endophthalmitis was reported in two eyes, where vitreous taps were culture‐positive for *Staphylococcus warneri*. These eyes fully recovered their visual function assessed after endophthalmitis management by vitrectomy and intravitreal antibiotics. In one of these eyes, BCVA changed from 36 to 38 ETDRS letters after endophthalmitis. In the other eye, BCVA changed from 33 to 72 ETDRS letters after endophthalmitis. This eye underwent cataract surgery, contributing to the increase in BCVA following endophthalmitis.

Overall, the incidence rate of IOI and symptomatic IOP increase were almost equally distributed across the follow‐up times with no significant differences in incidence rate being observed (Figure 3). However, there was a tendency towards a higher proportion of symptomatic IOP increases at 3‐month follow‐up, but without reaching statistical significance (*p* = 0.29) (Figure 3). Additionally, there was no difference in risk of AEs between eyes that were switched to aflibercept 8 mg and treatment naïve eyes that received aflibercept 8 mg as first‐line therapy (*p* = 0.71).

**FIGURE 3:**
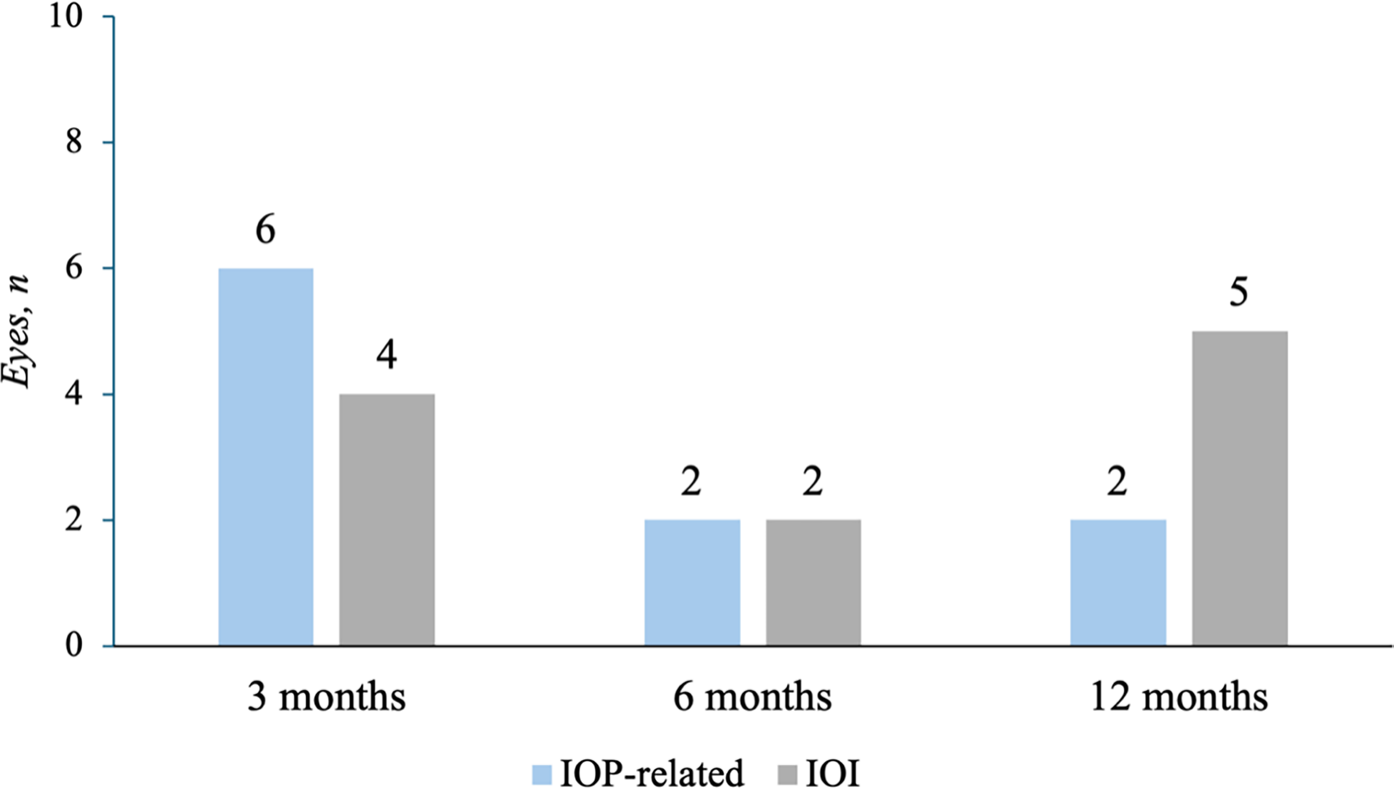
Distribution of symptomatic IOP‐related AE and IOI across 3‐, 6‐, or 12 months after treatment with aflibercept 8 mg. The results did not show any significantly higher risk of symptomatic IOP increase (*p* = 0.29) or IOI (*p* = 0.30) at any specific time point.

## 4 DISCUSSION

The current real‐world evidence on aflibercept 8 mg is predominantly based on reports on small sample sizes. The existing evidence on AEs associated with aflibercept 8 mg was recently evaluated in a systematic review combined with a meta‐analysis of a total of 9481 injections (Rebsdorf et al., 2025). Our study of 8675 injections is the largest real‐world study to date and a meaningful contribution to the generation of safety data for aflibercept 8 mg. Our study provides real‐world insights on the safety profile of aflibercept 8 mg over a 12‐month period, which is a meaningful follow‐up interval, compared with existing real‐world evidence (Rebsdorf et al., 2025). A total of 2.88 AEs per 1000 injections were observed, reflecting a low AE incidence rate similar to previous reports on other anti‐VEGF agents (Korva‐Gurung et al., 2023; Ramos et al., 2021; Van Der Reis et al., 2011). The low incidence rate of serious AEs also aligns with safety data of pulsar (Lanzetta et al., 2024). A similarly low incidence rate of AEs has also been reported across a range of other clinical trials (Korobelnik et al., 2025; Lanzetta et al., 2024; Stahl et al., 2024; Wykoff et al., 2023) and real‐world studies (Abu Ishkheidem et al., 2025; Musadiq et al., 2025; Wojciechowski et al., 2025). A recent real‐world study on 132 eyes reported no differences in safety outcomes between aflibercept 2‐ and 8 mg for the management of nAMD and diabetic macular edema (DME) (Anakwenze et al., 2025). A systematic review by Wojciechowski et al. (2025) established no significant differences in safety outcomes when comparing aflibercept 8 mg to aflibercept 2 mg, faricimab, ranibizumab, and bevacizumab. Additionally, our study did not find an increased risk of AEs between pre‐treated and treatment naïve eyes, suggesting that prior anti‐VEGF therapy did not impact the risk of AEs.

### 4.1 Intraocular inflammation (IOI)

The most frequent AE was IOI. In our cohort, we observed an incidence rate of 1.27 per 1000 injections (*n* = 11), which corresponds to 0.13% of the included eyes. A similar result was obtained in a real‐world study of 181 eyes by Abu Ishkheidem et al. (2025), where IOI was reported in 0.2% per injection among a total of 415 aflibercept 8 mg injections. Overall, the findings of our study do not raise concerns about severe IOI, as 10 out of 11 cases were managed successfully with topical dexamethasone eyedrops. Furthermore, our data did not demonstrate a post‐IOI change in BCVA, suggesting the IOI cases were generally mild. Our findings align with other real‐world studies reporting mild IOI following the administration of aflibercept 8 mg (Binder et al., 2025; Cattaneo et al., 2025; Dwomoh et al., 2025). A systematic review by Rebsdorf et al. (2025) reported an IOI incidence rate of 9.26 per 1000 injections in real‐world studies and 1.45 per 1000 injections in the pulsar, photon, and candela trials combined. As clinical trials are known to apply exclusion criteria related to IOI (Arnold‐Vangsted et al., 2025), it is surprising that the IOI incidence rate of our real‐world cohort was lower than the combined incidence of the clinical trials. However, patients were not systematically examined at every visit for IOI after injections in our cohort unless they reported symptoms. This is reflected in the delayed detection of IOI, which occurred in an average of 19 days after the intravitreal injections. Additionally, the systematic review by Rebsdorf et al. (2025) reported an incidence rate of retinal vasculitis of 6.18 per 1000 injections. While retinal vasculitis was not suspected in any of the IOI cases of our cohort, it is a limitation that evaluation by fluorescein/indocyanine green angiography was not performed, as this would only be performed upon relevant clinical suspicion based on fundoscopy and OCT in our clinic. In the IOI cases of our study, there were no funduscopic findings that raised suspicion of concurrent retinal vasculitis.

There is no indication that the incidence rate of IOI observed in our study was higher than for aflibercept 2 mg. In a study similar to ours, (Kim et al., 2015) previously reported an IOI incidence rate of 5.5 per 1000 injections for aflibercept 2 mg.

The exact mechanism of IOI associated with aflibercept 8 mg remains incompletely understood (Hoffmann et al., 2024), but the high molecular dose is considered a plausible explanation. This hypothesis is supported by case reports with no IOI on aflibercept 2 mg, while aflibercept 8 mg resulted in IOI (Sato et al., 2025). A type III hypersensitivity reaction involving anti‐drug antibodies or inflammatory reactions caused by vascular endothelial cell damage has been discussed (Kusuhara et al., 2022; Sharma et al., 2020, 2022).

Unlike the clinical trials, our study did not assess IOI routinely. IOI was assessed if the patients complained about symptoms and at routine follow‐up visits. Our study provides insights on IOI requiring active management, but subclinical IOI may potentially be underreported as a result of the retrospective evaluation.

### 4.2 Intraocular pressure (IOP)‐related adverse events (AEs)

In this study, symptomatic IOP was reported in 10 eyes, with blurred vision being the most reported symptom. Furthermore, eight out of 10 eyes were initiated on IOP‐lowering intervention. IOP‐lowering medications were prescribed in most cases and only a few eyes underwent volume reduction of aflibercept 8 mg from 0.07 to 0.05 mL. Volume reduction is rarely performed in clinical practice, and only a small subset of eyes (*n* = 6) received this intervention. This differs from previous studies, where IOP‐lowering intervention was not required (Gartaganis et al., 2025; Musadiq et al., 2025; Palm et al., 2025). The number of eyes experiencing symptomatic IOP increase in our cohort was relatively low. An observed trend showed that most IOP‐related AEs occurred within the first 3 months after initiating aflibercept 8 mg, with a decreasing incidence after 6 and 12 months. For other anti‐VEGF agents, a higher risk of IOP elevation was observed following repeated intravitreal injections (Daka et al., 2023; Lereuil et al., 2017; Maruyama‐Inoue et al., 2021; Zhou et al., 2016). In addition, repeated intravitreal injections have been linked to irreversible damage affecting the optic nerve head and retinal nerve fibre layer (De Vries et al., 2020; Gómez‐Mariscal et al., 2019; Viggiano et al., 2024). However, several studies have observed no significant difference in IOP when comparing aflibercept 2 and 8 mg (Korobelnik et al., 2025; Lu et al., 2025; Paris et al., 2025). Transient IOP increase is a well‐known phenomenon following intravitreal injections (Colizzi et al., 2025; LoBue et al., 2023; Paris et al., 2025) with glaucomatous eyes being more susceptible to developing IOP increase (Colizzi et al., 2025; LoBue et al., 2023; Yamamoto et al., 2025). Unlike the clinical trials, post‐injection IOP measurement was not performed routinely. While our study provides insights into the incidence of symptomatic IOP increase following the administration of aflibercept 8 mg, asymptomatic IOP increases may be underreported. IOP was assessed using iCare tonometry, which is associated with a slight overestimation of IOP of 1.4–2.0 mmHg compared to Goldmann applanation tonometry. Although the iCare tonometry could potentially overestimate IOP, the incidence of symptomatic IOP increase was very low. In the current series, there was no difference between glaucomatous eyes versus non‐glaucomatous eyes in terms of symptomatic IOP increase. This finding was surprising, given the large sample size of glaucomatous eyes (*n* = 139) of our cohort, and thereby suggests a low risk of symptomatic IOP increase in glaucomatous eyes. Our results indicate that precautions may primarily be required for eyes that have undergone filtration surgery and hyperopic glaucomatous eyes with previous angular closure.

### 4.3 Other adverse events (AEs)

The higher volume delivered for aflibercept 8 mg has raised concerns over retinal vascular occlusion. Despite more than 8500 administered injections, no cases of retinal vascular occlusion were observed, which is consistent with the safety data of the clinical trials (Lanzetta et al., 2024; Stahl et al., 2024; Wykoff et al., 2023). pulsar (Lanzetta et al., 2024) and candela (Wykoff et al., 2023) reported no occurrence of bacterial endophthalmitis. We identified two cases of bacterial endophthalmitis in our cohort, which is comparable to a previous report on vial‐administered aflibercept 8 mg (Papproth et al., 2025).

### 4.4 Strengths and limitations

Our study was conducted exclusively on nAMD patients, but AEs may have a different presentation in eyes with DME or retinal vein occlusion (RVO). IOP dynamics may potentially have a different role in eyes with RVO, given the fact that glaucoma is a known risk factor of RVO, especially central retinal vein occlusion (Chou et al., 2025; Kim et al., 2021; Yin et al., 2019). AEs were only registered if stated in the medical records. Since the medical records included were obtained at a tertiary retina clinic, some AEs may have been observed by an ophthalmologist outside the hospital setting without being registered in hospital records. The mean age of our population was substantially higher than the mean in pulsar (Lanzetta et al., 2024), and this difference in mean age may potentially impact the risk profiles of the population.

## 5 CONCLUSION

Our study identified a low incidence rate of AEs following the administration of aflibercept 8 mg. IOI cases were generally mild and successfully managed with topical dexamethasone eyedrops except for one case that required vitrectomy. Despite a large number of glaucomatous eyes in the cohort, our data did not demonstrate a statistically significant higher proportion of symptomatic IOP increases in glaucomatous eyes versus non‐glaucomatous eyes. The incidence rate of bacterial endophthalmitis was low and consistent with previous reports on aflibercept 8 mg real‐world evidence. No case of retinal vasculitis or retinal vascular occlusion was observed.

## AUTHOR CONTRIBUTIONS

Mary Graciya Karunainathan, Celine Lee, Lasse Jørgensen Cehofski, Marie Ørskov had full access to all of the data in the study and take responsibility for the integrity of the data and the accuracy of the data analysis. Lasse Jørgensen Cehofski, Yousif Subhi: Study concept and design. Mary Graciya Karunainathan, Lasse Jørgensen Cehofski, Marie Ørskov, Kaweh Daniel Zohalinejad, Mads Odgaard Mæng, Nanna Würtz Fabrin, Sigurrós Jónsdóttir, Anne Kjærsgaard Hassing, Søren Kveiborg Yde: Acquisition, analysis, or interpretation of data. Mary Graciya Karunainathan and Lasse Jørgensen Cehofski: Drafting of the manuscript. Henrik Vorum, Lasse Jørgensen Cehofski, Marie Ørskov, Yousif Subhi, Charles C. Wykoff: Critical revision of the manuscript for important intellectual content. Mary Graciya Karunainathan, Marie Ørskov: Statistical analysis. Celine Lee, Lasse Jørgensen Cehofski: Administrative, technical, or material support. Lasse Jørgensen Cehofski, Marie Ørskov, Yousif Subhi, Charles C. Wykoff: Study supervision.

## FUNDING INFORMATION

The authors have nothing to report.

## CONFLICT OF INTEREST STATEMENT

Lasse Jørgensen Cehofski: AbbVie (speaker honorarium, Advisory Board), Roche (EURETINA Mentorship Programme, Advisory Board), Yousif Subhi: Roche (speaker honorarium); Henrik Vorum: Bayer (Advisory Board), Roche (Advisory Board). Charles C. Wykoff: Consultancy for the following companies: 4DMT, AbbVie, Acuta, ADARx, Adverum, Aerie, Alcon, Alkeus, Allgenesis, AMC Sciences, ANI, Annexon, Apellis, Arrowhead, Ascidian, Ashvattha, Astellas, Aviceda, Bausch + Lomb, Bayer, Biocryst, Bionic Vision, Boehringer Ingelheim, Chengdu Kanghong, Curacle, Emmecell, Eyconis, EyeBiotech, EyePoint, Foresite, Frontera, Genentech, Harrow, IACTA, InGel, Janssen, Kato, Kiora, Kodiak, Kowa, Merck, MingSight, Nanoscope, Neurotech, NGM, Novartis, Ocular Therapeutix, OcuTerra, Ollin, ONL, Opthea, Opus, Osanni, Oxular, Palatin, Perceive Bio, Perfuse, Ray, Regeneron, RegenXBio, RetinAI, Roche, Samsung Bioepis, Sanofi, Santen, SciNeuro, Skyline, Stealth, Surrozen, Suzhou Raymon, Sylentis, TCG Crossover, TCG‐Soleil, THEA, Therini, Topcon, VH401, Visgenx, Zeiss, ZipBio.
